# A feature selection method for classification within functional genomics experiments based on the proportional overlapping score

**DOI:** 10.1186/1471-2105-15-274

**Published:** 2014-08-11

**Authors:** Osama Mahmoud, Andrew Harrison, Aris Perperoglou, Asma Gul, Zardad Khan, Metodi V Metodiev, Berthold Lausen

**Affiliations:** Department of Mathematical Sciences, University of Essex, Wivenhoe Park, CO4 3SQ Colchester, UK; School of Biological Sciences/Proteomics Unit, University of Essex, Wivenhoe Park, CO4 3SQ Colchester, UK; Department of Applied Statisitcs, Helwan University, Cairo, Egypt

**Keywords:** Feature selection, Gene ranking, Microarray classification, Proportional overlap score, Gene mask, Minimum subset of genes

## Abstract

**Background:**

Microarray technology, as well as other functional genomics experiments, allow simultaneous measurements of thousands of genes within each sample. Both the prediction accuracy and interpretability of a classifier could be enhanced by performing the classification based only on selected discriminative genes. We propose a statistical method for selecting genes based on overlapping analysis of expression data across classes. This method results in a novel measure, called proportional overlapping score (POS), of a feature’s relevance to a classification task.

**Results:**

We apply POS, along‐with four widely used gene selection methods, to several benchmark gene expression datasets. The experimental results of classification error rates computed using the Random Forest, *k* Nearest Neighbor and Support Vector Machine classifiers show that POS achieves a better performance.

**Conclusions:**

A novel gene selection method, POS, is proposed. POS analyzes the expressions overlap across classes taking into account the proportions of overlapping samples. It robustly defines a mask for each gene that allows it to minimize the effect of expression outliers. The constructed masks along‐with a novel gene score are exploited to produce the selected subset of genes.

**Electronic supplementary material:**

The online version of this article (doi:10.1186/1471-2105-15-274) contains supplementary material, which is available to authorized users.

## Background

Microarray technology, as well as other high‐throughput functional genomics experiments, have become a fundamental tool for gene expression analysis in recent years. For a particular classification task, microarray data are inherently noisy since most genes are irrelevant and uninformative to the given classes (phenotypes). A main aim of gene expression analysis is to identify genes that are expressed differentially between various classes. The problem of identification of these discriminative genes for their use in classification has been investigated in many studies [1]‐[9]. Assessment of maximally selected genes or prognostic factors ‐ equivalently selected by the minimum p‐value approach ‐ have been discussed in [10, 11] using data from clinical cancer research and gene expression. The solution is to use an appropriate multiple testing framework, but obtaining study or experiment optimised cut‐points for selected genes make comparison with other studies and results difficult.

A major challenge is the problem of dimensionality; tens of thousands of genes’ expressions are observed in a small number, tens to few hundreds, of samples. Given an input of gene expression data along‐with samples’ target classes, the problem of gene selection is to find among the entire dimensional space a subspace of genes that best characterizes the response target variable. Since the total number of subspaces with dimension not higher than *r* is , where *P* is the total number of genes, it is hard to search the subspaces exhaustively [8]. Alternatively, various search schemes have been proposed e.g., best individual genes [9], Max‐Relevance and Min‐Redundancy based approaches [8], Iteratively Sure Independent Screening [12] and MaskedPainter approach [7]. Identification of discriminative genes can be based on different criteria including: p‐values of statistical tests e.g. t‐test or Wilcoxon rank sum test [10, 11]; ranking genes using statistical impurity measures e.g. information gain, gini index and max minority [9]; analysis of overlapping expressions across different classes [6, 7].

A way to improve prediction accuracy, as well as interpretation of the biological relationship between genes and the considered clinical outcomes, is to use a supervised classification based on expressions of discriminative genes identified by an effective gene selection technique. This procedure of pre‐selection of informative genes also helps in avoiding overfitting and building a faster model by providing only the features that contribute most to the considered classification task. However, a search for the subset of informative genes presents an additional layer of complexity in the learning process. In depth reviews of feature selection methods in the microarray domain can be found in [13].

One of the differences among various feature selection procedures is the way they perform the search in the feature space. Three categories of feature selection methods can be distinguished: wrapper, embedded and filter methods.

*Wrapper methods* evaluate gene subsets using a predictive model which is run on the dataset partitioned into training and testing sets. Each gene subset is used with training dataset to train the model, which is then tested on the test set. Calculating a model prediction error from the test set gives a score for that gene subset. The gene subset with the highest evaluation is selected as the final set on which to run this particular model. The wrapper methods are computationally expensive since they need a new model to be fitted for each gene subset. Genetic algorithm based feature selection techniques are representative examples for wrapper methods [13].

*Embedded methods* perform feature selection search as part of the model construction process. They are less computationally expensive than the wrapper methods. An example of this category is a classification tree based classifier [14].

*Filter methods* assess genes by calculating a relevant score for each gene. The low‐relevant genes are then removed. The selected genes may then be used to serve classification via many types of classifiers. Gene selection filter‐based methods can scale easily to high‐dimensional datasets since they are computationally simple and fast compared with the other approaches. Various examples for filter‐based approaches have been proposed in earlier papers [2, 3, 15]‐[17]. Filtering methods can introduce a measure for assessing importance of genes [2, 15, 18, 19], present thresholds by which informative genes are selected [3] or fit a statistical model to expression data in order to identify the discriminative features [16, 17]. A measure named ‘relative importance’, proposed by Draminski et al. [2], is used to assess genes and to identify informative ones based on their contribution in the process of classifying samples when large number of classification trees have been constructed. The contribution of a particular gene to the relative importance measure is defined by a weighted scale of the overall number of splits made on that gene in all constructed trees. The authors of [2] use decision tree classifiers for measuring the genes’ relative importance, not for the aim of fitting classification rules. Ultsch et al. [15] propose an algorithm, called ‘PUL’, in which the differentially expressed genes are identified based on a measure for retrieval information named PUL‐score. Ding et al. [18] propose a framework, named ‘minimal redundancy maximal relevance (mRMR)’ based on a series of intuitive measures of relevance, to the response target, and redundancy, between genes being selected. De Jay et al. [19] developed an R package, named ‘mRMRe’, by which an ensemble version of mRMR has been implemented. The authors of [19] use two different strategies to select multiple features sets, rather than a single set, in order to mitigate the potential effect of the low sample‐to‐dimensionality ratio on the stability of the results. Marczyk et al. [3] propose an adaptive filter method based on the decomposition of the probability density function of gene expression means or variances into a mixture of Gaussian components. They determine thresholds to filter genes via tuning the proportion between the pools sizes of removed and retained genes. Lu et al. [16] propose another criterion to identify the informative genes in which principle component analysis has been used to explore the sources of variation in the expression data and to filter out genes corresponding to components with less variation. Tallon et al. [17] use factor analysis models rather than principle component analysis to identify informative genes. A comparison between some algorithms for identifying informative genes in microarray data can be found in [15, 20].

Analyzing the overlap between gene expression measures for different classes can be another important criterion for identifying discriminative genes which are relevant to the considered classification task. This strategy utilities the information given by sample classes as well as expression data for detection of the differentially expressed genes between target classes. A classifier can then use these selected genes to enhance its classification performance and prediction accuracy. A procedure specifically designed to select genes based on their overlapping degree across different classes was recently proposed [6]. This procedure, named Painter’s feature selection method, proposes a simplified version of a measure calculating an overlapping score for each gene. For binary class situations, this score estimates the overlapping degree between both classes taking into account only one factor i.e., length of the interval of overlapping expressions. It has been defined to provide higher scores for longer overlapping intervals. Genes are then ranked in ascending order according to their scores. This simplified measure has been extended by Apiletti et al. [7] using another factor, i.e. the number of overlapped samples, in the analysis. The authors of [7] characterize each gene by means of a *gene mask* that represents the capability of a gene to unambiguously assign training samples to their correct classes. Characterization of genes using training sample masks with their overlapping scores allow the detection of the minimum set of genes that provides the best classification coverage on training samples. A final gene set is then provided by combining the minimum gene subset with the top ranked genes according to the overlapping score. Since gene masks, proposed by [7], are defined based on the range of the training expression intervals, a caveat of this technique is that the construction of gene masks could be affected by outliers.

Biomedical researchers may be interested in identifying small sets of genes that could be used as genetic markers for diagnostic purposes in clinical researches. This typically involves obtaining the smallest possible subset of genes that can still provide a good predictive performance, whilst removing redundant ones [21]. We propose a procedure serving this goal, by which the minimum set of genes is selected to yield the best classification accuracy on a training set avoiding the effects of outliers.

In this article, we propose a new gene selection method, called POS, that can be described as follows:

1. POS utilizes the interquartile range approach to robustly detect the minimum subset of genes that maximizes the correct assignment of training samples to their corresponding classes i.e., the minimum subset that can yield the best classification accuracy on a training set avoiding the effects of outliers.
2. A new filter‐based technique which ranks genes according to their predictive power in terms of the overlapping degree between classes is proposed. In this context, POS presents a novel generalized version, called *POS* score, of the overlapping score (OS) measure, proposed in [7].
3. POS provides genes categorization into the target class labels based on their relative dominant classes i.e., POS assigns each gene to the class label that has the highest proportion of correctly assigned samples relative to class sizes.

In a benchmarking experiment, the classification error rates of the Random Forest (RF) [22], *k* Nearest Neighbor (*k*NN) [23], and Support Vector Machine (SVM) [24] classifiers demonstrate that our approach achieves a better performance than several other widely used gene selection methods.

The paper is organized as follows. Section ‘Methods’ explains the proposed method. The results of our approach are compared with some other feature selection techniques in section ‘Results and discussion’. Section ‘Conclusion’ concludes the paper and suggests future directions.

## Methods

### POS approach for binary class problems

Microarray data are usually presented in the form of a gene expression matrix, *X*=[*x*_*ij*_], such that *X*∈ℜ^*P*×*N*^ and *x*_*ij*_ is the observed expression value of gene *i* for tissue sample *j* where *i*=1, …, *P* and *j*=1, …, *N*. Each sample is also characterized by a target class label, *y*_*j*_, representing the phenotype of the tissue sample being studied. Let  be the vector of class labels such that its *j*th element, *y*_*j*_, has a single value *c* which is either 1 or 2.

**Figure 1:**
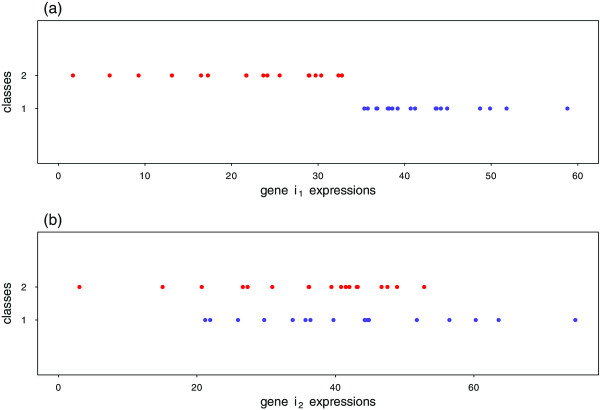
**An example for two different genes with different overlapping pattern.** Expression values of two different genes (*i*_1_, *i*_2_) each of which with 36 samples belonging to 2 classes, 18 samples for each class: **(a)** expression values of gene *i*_1_, **(b)** expression values of gene *i*_2_.

Analyzing the overlap between expression intervals of a gene for different classes can provide a classifier with an important aspect of a gene’s characteristic. The idea is that a certain gene *i* can assign samples (patients) to class *c* because their gene *i* expression interval in that class is not overlapping with gene *i* intervals of the other class. In other words, gene *i* has the ability to correctly classify samples for which their gene *i* expressions fall within the expression interval of a single class. For instance, Figure 1a presents expression values of gene *i*_1_ with 36 samples belonging to two different classes. It is clear that gene *i*_1_ is relevant for discriminating samples between the target classes, because their values are falling in non‐overlapping ranges. Figure 1b, on the other hand, shows expression values for another gene *i*_2_, which looks less useful for distinguishing between these target classes, because their expression values have a highly overlapping range.

POS initially exploits the interquartile range approach to robustly define gene masks that report the discriminative power of genes with a training set of samples avoiding outlier effects. Then, two measures are assigned for each gene: proportional overlapping score (*POS*) and relative dominant class (*RDC*). Analogously to [7] these two novel measures are exploited in the ranking phase to produce the final set of ranked genes. *POS* is a gene relevance score that estimates the overlapping degree between the expression intervals of both given classes taking into account three factors: (1) length of overlapping region; (2) number of overlapped samples; (3) the proportion of classes’ contribution to the overlapped samples. The latter factor is the incentive for the name we gave to our procedure, Proportional Overlapping Scores (POS). The relative dominant class (*RDC*) of a gene is the class that has the highest proportion, relative to class sizes, of correctly assigned samples.

### Definition of core intervals

**Figure 2:**
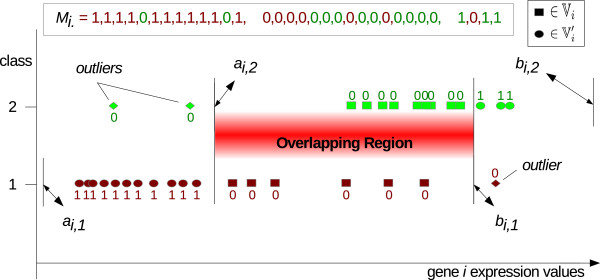
**Core intervals with gene mask.** An example for core expression intervals of a gene with 18 and 14 samples belonging to class 1, in red colour, and class 2, in green colour, respectively with its associated mask elements. Elements of the overlapping samples set and non‐overlapping samples set are highlighted by squares and circles respectively.

For a certain gene *i*, by considering the expression values *x*_*ij*_ with a class label *c*_*j*_ for each sample *j*, we can define two expression intervals, one for each class, for that gene. The *c*th class interval for gene *i* can be defined in the form:
1

such that:
2

where ,  and *IQR*^(*i*,*c*)^ denote the first, third empirical quartiles, and the interquartile range of gene *i* expression values for class *c* respectively. Figure 2 shows the potential effect of expression outliers on extending the underlying intervals, if the range of training expressions are considered. Based on the defined core intervals, we present the following definitions: ***Non‐outlier samples set***, , for gene *i* is defined as the set of samples whose expression values fall inside their own target classes core interval. This set can be expressed as:
3

where *c*_*j*_ is the correct class label for sample *j*. ***Total core interval***, *I*_*i*_, for gene *i* is given by the region between the global minimum and global maximum boundaries of core intervals for both classes. It is defined as:
4

such that: *a*_*i*_=*min*{*a*_*i*,1_, *a*_*i*,2_}, *b*_*i*_=*max*{*b*_*i*,1_, *b*_*i*,2_}, where *a*_*i*,*c*_, *b*_*i*,*c*_ respectively represent the minimum and maximum boundaries of core interval, *I*_*i*,*c*_, of gene *i* with target class *c*=1, 2, (see equations 1 and 2). ***The overlap region***, , for gene *i* is defined as the interval yielded by the intersection between core expression intervals of both target classes. It can be addressed as:
5

***Overlapping samples set***, , for gene *i* is the set containing the samples whose expression values fall within the overlap interval , defined in the overlap region definition (see equation 5). The overlapping sample set can be defined as:
6

where  represents the non‐overlapping samples set which is defined as follows. ***Non‐overlapping samples set***, , for gene *i* is defined as the set consisting of elements of , defined in equation 3, whose expression values don’t fall within the overlap interval , defined in equation 5. In this way, we can define this set as:
7

For convenience, 〈*I*〉 notation is used with interval *I* to represent its length while |.| notation is used with set {.} to represent its size.

### Gene masks

For each gene, we define a mask based on its observed expression values and constructed core intervals presented in subsection ‘Definition of core intervals’. Gene *i* mask reports the samples that gene *i* can unambiguously assign to their correct target classes, i.e. the non‐overlapping samples set . Thus, gene masks can represent the capability of genes to classify correctly each sample, i.e. it represents a gene’s classification power. For a particular gene *i*, element *j* of its mask is set to 1 if the corresponding expression value *x*_*ij*_ belongs only to core expression interval  of the single class *c*_*j*_, i.e. if sample *j* is a member of the set . Otherwise, it is set to zero.

We define the gene masks matrix *M*=[*m*_*ij*_] in which the mask of gene *i* is presented by *M*_*i*._(the *i*th row of *M*) such that gene mask element *m*_*ij*_ is defined as:
8

Figure 2 shows the constructed core expression intervals *I*_*i*,1_ and *I*_*i*,2_ associated with a particular gene *i* along‐with its gene mask. The gene mask presented in this figure is sorted corresponding to the observations ordered by increasing expression values.

### The proposed *POS*measure and relative dominant class assignments

**Figure 3:**
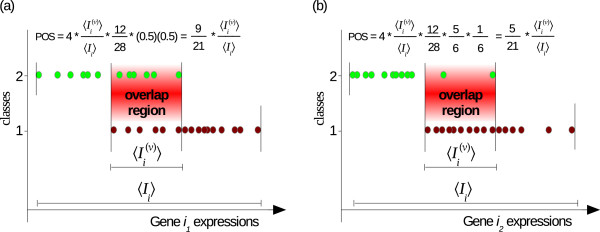
**Illustration for overlapping intervals with different proportions.** Examples for expression values of 2 genes distinguishing between 2 classes: **(a)** gene *i*_1_ has overlapping samples distributed as 1:1, **(b)** gene *i*_2_ has its overlapping samples distributed as 5:1 for class1:class2.

A novel overlapping score is developed to estimate the overlapping degree between different expression intervals. Figures 3a and 3b represent examples of 2 different genes, *i*_1_ and *i*_2_, with the same length of overlap interval, , length of total core interval, , and total number of overlapped samples, . These figures demonstrate that performing the ordinary overlapping scores, proposed in earlier papers [6, 7], result in the same value for both genes. But, there is an element which differs in those examples and it may also affect the overlap degree between classes. This element is the distribution of overlapping samples by classes. Gene *i*_1_ has six overlapped samples from each class, whereas gene *i*_2_ has ten and two overlapping samples from class 1 and 2 respectively. By taking this status into account, gene *i*_2_ should be reported to have less overlap degree compared to gene *i*_1_. In this article, we develop a new score, called proportional overlapping score (*POS*), that estimates the overlapping degree of a gene taking into account this element, i.e. proportion of each class’s overlapped samples to the total number of overlapping samples.

*POS* for a gene *i* is defined as:
9

where *θ*_*c*_ is the proportion of class *c* samples among overlapping samples. Hence, *θ*_*c*_ can be defined as:
10

where  represent set of overlapping samples belonging to class *c*, . According to equation 9, values of *POS* measure are  and  for genes *i*_1_ and *i*_2_ in Figures 3a and 3b respectively.

Larger overlapping intervals or higher numbers of overlapping samples results in an increasing *POS* value. Furthermore, as proportions *θ*_1_ and *θ*_2_ get closer to each other, the *POS* value increases. The most overlapping degree for a particular gene is achieved when *θ*_1_=*θ*_2_=0.5 while the other two factors are fixed. We include the multiplier “4” in equation 9 to scale *POS* score to be within the closed interval [0,1]. In this way, a lower score denotes gene with higher discriminative power.

Once the gene mask is defined and *POS* index is computed, we assign each gene to its relative dominant class (*RDC*). *RDC* for gene *i* is defined as follows:
11

where  is the set of class *c* samples . Note that , while *m*_*ij*_ is the *j*th mask element of gene *i* (see equation 8). *I*(*m*_*ij*_=1) represents an indicator which sets to 1 if *m*_*ij*_=1, otherwise it sets to zero.

In this definition, the samples that belong to the set  categorized into their target classes are only considered for each class. These samples are the ones that the gene could unambiguously assign to their target classes. According to our gene mask definition (see equation 8) they are the samples with 1 bits in the corresponding gene mask. Afterwards, the proportion of the class’s samples to its total sample size has been evaluated. The class with the highest proportion is the relative dominant class of the gene. Ties are randomly distributed on both classes. Genes are assigned to their *RDC* in order to associate each gene with the class it is more able to distinguish. As a result, the number of selected genes could be balanced per class at our final selection process. The relative evaluation for detecting the dominant class can avoid the misleading assignment due to unbalanced class sizes distribution effects.

### Selecting minimum subset of genes

Selecting a minimum subset of genes is one of the POS method stages in which the information provided by the constructed gene masks and the *POS* scores are analyzed. This subset is designated to be the minimum one that correctly classify the maximum number of samples in a given training set, avoiding the effects of expression outliers. Such a procedure allows disposing of redundant information e.g., genes with similar expression profiles.

Baralis et al. [25] have proposed a method that is somewhat similar to our procedure for detecting a minimum subset of genes from microarray data. The main differences are that [25] use the expression range to define the intervals which are employed for constructing gene masks, and then apply a set‐covering approach to obtain the minimum feature subset. The same technique is performed by [7] to get a minimum gene subset using a greedy approach rather than the set‐covering.

Let  be a set containing all genes (i.e., ). Also, let  be its aggregate mask which is defined as the logical disjunction *(logic OR)* between all masks corresponding to genes that belong to the set. It can be expressed as follows:
12

Our objective is to search for the minimum subset, denoted by , for which  equals to the aggregate mask of the set of genes, . In other words, our minimum set of genes should satisfy the following statement:
13

A modified version of the greedy search approach used by [7] is applied. The pseudo code of our procedure is reported in Algorithm 1. Its inputs are the matrix of gene masks, *M*; the aggregate mask of genes, ; and *POS* scores. It produces the minimum set of genes, , as output.


At the initial step (*k*=0), we let  and  (lines 2, 3); where  is the aggregate mask of the set , while **0**_*N*_ is a vector of zeros with the length *N*. Then, at each iteration, *k*, the following steps are performed:

1. The gene(s) with the highest number of mask bits set to 1 is (are) chosen to form the set  (line 6). This set could not be empty as long as the loop condition is still satisfied, i.e. . Under this condition, our selected genes don’t cover yet the maximum number of samples that should be covered by our target gene set. Note that our definition for gene masks allows  to report in advance which samples should be covered by the minimum subset of genes. Therefore, there would be at least one gene mask which has at least one bit set to 1 if that condition is to hold.
2. The gene with the lowest *POS* score among genes in , if there are more than one, is then selected (line 7). It is denoted by *g*_*k*_.
3. The set  is updated by adding the selected gene, *g*_*k*_ (line 8).
4. All gene masks are also updated by performing the logical conjunction (*logic AND*) with negated aggregate mask of set  (line 10). The negated mask  of the mask  is the one obtained by applying logical negation (logical complement) on this mask. Consequently, the bits of ones corresponding to the classification of still uncovered samples are only considered. Note that  represents updated mask of gene *i* at the *k*th iteration such that  is its original gene mask whose elements are computed according to equation 8.
5. The procedure is successively iterated and ends when all gene masks have no one bits anymore, i.e. the selected genes cover the maximum number of samples. This situation is accomplished iff .

Thus, this procedure detects the minimum set of genes required to provide the best classification coverage for a given training set. In addition, genes are descendingly ordered by number of 1 bits within the minimum set, .

### Final gene selection

The *POS* score alone can rank genes according to their overlapping degree, without taking into account the class that has more correctly assigned samples by each gene (which can be addressed as the dominant class of that gene). Consequently, high‐ranked genes may all have an ability to only correctly classify samples belonging to the same class. Such a case is more likely to happen in situations with unbalanced class‐size distributions. As a result, a biased selection could result. Assigning the dominant class on a relative basis, as proposed in subsection ‘The proposed *POS* measure and relative dominant class assignments’, and taking these assignments into account during the gene ranking process allows us to overcome this problem.

Therefore, the gene ranking process is performed by considering both *POS* scores and *RDC*. Within each relative dominant class *c* (where *c*=1,2), all genes that have not been chosen in the minimum set, , and whose *RDC*=*c* are sorted by an increasing order of *POS* values. Now, we have two disjoint groups (one for each class) of ranked genes. The topmost gene is selected from each group in a round‐robin fashion to compose the gene ranking list.

The minimum subset of genes, presented in subsection ‘Selecting minimum subset of genes’, is extended by adding the top *ν* ranked genes in the gene ranking list, where *ν* is the required number extending the minimum subset up to the total number of requested genes, *r*, which is an input of the POS method set by the user. The resulting final set includes the minimum subset of genes regardless of their *POS* values, because these genes allow the considered classifier to correctly classify the maximum number of training samples.

The pseudo code of the Proportional Overlapping Scores (POS) method is reported in Algorithm 2.


## Results and discussion

For evaluating different feature selection methods, one can assess the accuracy of a classifier applied after the feature selection process. Thus, the classification is based only on selected gene expressions. Such an assessment can verify the efficiency of identification of discriminative genes. Jirapech and Aitken [26] have analyzed several gene selection methods available in [9] and have shown that the gene selection method can have a significant impact on a classifier’s accuracy. Such a strategy has been applied in many studies including [7] and [8].

In this article, our experiment is conducted using eleven gene expression datasets in which the POS method is validated by comparison with five well‐known gene selection techniques. The performance is evaluated by obtaining the classification error rates from three different classifiers: Random Forest (RF); *k* Nearest Neighbor (*k*NN); Support Vector Machine (SVM).

Table 1 summarizes the characteristics of the datasets. The estimated classification error rate is based on the Random Forest classifier with the full set of features, without pre‐selection, using 50 repetitions of 10‐fold cross validation. Eight of the datasets are bi‐class, while three, i.e. Srbct, GSE14333 and GSE27854, are multi‐classes. The two classes with topmost number of samples are only considered for the Srbct data, while the remaining classes are ignored, since we are interested only in binary classification analysis. For the GSE14333 data, patients with colorectal cancer of I and II tumor ‘Union Internationale Contre le Cancer (UICC)’ stages are combined in a single class representing non‐invasive tumors, against patients with stage III, which represents invasive tumors. Whereas for the GSE27854 data, a class composed of colorectal cancer patients with UICC stages I and II is defined against another class involving patients with III and IV stages. All datasets are publicly available, see section ‘Availability of supporting data’.

**Table 1:** **Description of used gene expression datasets**

***Dataset***	***Genes***	***Samples***	***Class‐sizes***	***Est. Error***	***Source***
Leukaemia	7129	72	47/25	0.049	[27]
Breast	4948	78	34/44	0.369	[28]
Srbct	2308	54	29/25	0.0008	[29]
Prostate	10509	102	52/50	0.088	[29]
All	12625	128	95/33	0.000	[30]
Lung	12533	181	150/31	0.003	[31]
Carcinoma	7457	36	18/18	0.027	[32]
GSE24514	22215	49	34/15	0.0406	[33]
GSE4045	22215	37	29/8	0.2045	[34]
GSE14333	54675	229	138/91	0.4141	[35]
GSE27854	54675	115	57/58	0.4884	[36]

Fifty repetitions of 10‐fold cross validation analysis were performed for each combination of dataset, feature selection algorithm, and a given number of selected genes, up to 50, with the considered classifiers. Random Forest is implemented using the R package ‘randomForest’ with its default parameters, i.e. ntree, mtry and nodesize are 500,  and 1 respectively. The R packages ‘class’ and ‘e1071’ are used to perform the *k* Nearest Neighbor and Support Vector Machine classifiers respectively. The parameter *k* for *k*NN classifier is chosen to be  rounded to the nearest odd number, where *N* is the total number of observations (tissue samples). For each experimental repetition, the split seed was changed while the same folds and training datasets were kept for all feature selection methods. To avoid bias, gene selection algorithms have been performed only on the training sets. For each fold, the best subset of genes has been selected according to the Wilcoxon Rank Sum technique (Wil‐RS), Minimum Redundancy Maximum Relevance (mRMR) method [8], MaskedPainter (MP) [7], Iteratively Sure Independent Screening (ISIS) [12], along‐with our proposed method. The expressions of the selected genes as well as the class labels of the training samples have then been used to construct the considered classifiers. The classification error rate on the test set is separately reported for each classifier and the average error rate over all the fifty repetitions is then computed. Due to limitations of the R package ‘mRMRe’ [19], mRMR selections could not be conducted for datasets having more than ‘46340’ features. Therefore, mRMR method is excluded from the analysis of the ‘GSE14333’ and ‘GSE27854’ datasets.

The compared feature selection methods are used commonly within the microarray data analysis domain. Apiletti et al. [7] demonstrate that the MaskedPainter method has outperformed many widely used gene selection methods available in [9]. The mRMR technique, proposed in [18], is intensively used in microarray data analysis e.g., [19, 37]. The ISIS feature selection method exploits the principle of correlation ranking with its ‘sure independence screening’ property showed in [38] to select a set of features based on an iterative process. In our experiment, the ISIS technique has been applied using the ‘SIS’ R package.

For large enough input feature sets, effective classifier algorithms may have more ability to mitigate the potential effects of noisy and uninformative features by focusing more on the informative ones. For instance, the Random Forest algorithm employs an embedded feature selection procedure that results in less reliance on uninformative input features. In other words, selecting a large number of features may allow a classifier to compensate for potential feature selection shortcomings. For the purpose of comparing the effectiveness of the considered feature selection techniques in improving the classification accuracy, the experiment is designed to focus on small sets of selected features, up to 50 genes.

**Figure 4:**
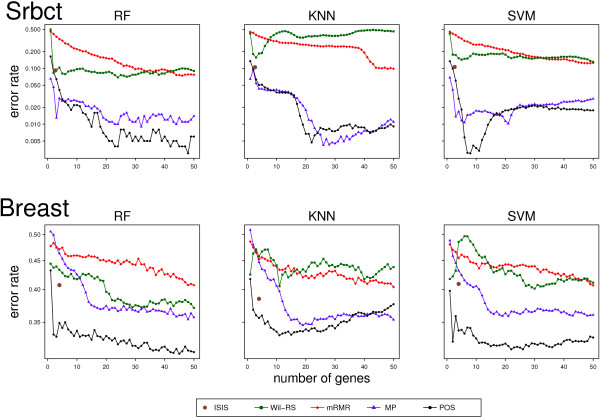
**Averages of classification error rates for ‘Srbct’ and ‘Breast’ datasets.** Average classification error rates for ‘Srbct’ and ‘Breast’ data based on 50 repetitions 10‐fold CV using ISIS, Wil‐RS, mRMR, MP and POS methods.

Tables 2 and 3 show the average classification error rates obtained by Wil‐RS, mRMR, MP and POS with RF, *k*NN and SVM classifiers on Leukaemia and GSE24514 datasets respectively. Each row provides the average classification error rate at a specific number of selected genes, reported in the first column. The aggregate average error value and the minimum error rate for each method with each classifier are provided in the last two rows. Average error rates yielded on the Breast and Srbct datasets using RF, *k*NN, and SVM classifiers are shown in Figure 4.

**Table 2:** **Average classification error rates yielded by Random Forest,** ***k*** **Nearest Neighbors and Support Vector Machine classifiers on ‘Leukaemia’ dataset over all the 50 repetitions of 10‐fold cross validation**

	RF				***k***NN				SVM			
N. genes	Wil‐RS	mRMR	MP	POS	Wil‐RS	mRMR	MP	POS	Wil‐RS	mRMR	MP	POS
1	0.126	0.211	0.015	**0.003**	0.141	0.220	0.019	**0.005**	0.133	0.238	0.022	**0.005**
2	0.083	0.197	0.017	**0.001**	0.110	0.195	0.059	**0.047**	0.099	0.197	0.053	**0.026**
3	0.068	0.185	0.020	**0.003**	0.086	0.198	**0.070**	0.073	0.078	0.198	0.064	**0.044**
4	0.044	0.180	0.016	**0.001**	0.082	0.194	0.076	**0.069**	0.068	0.178	0.070	**0.050**
5	0.043	0.168	0.015	**0.002**	0.077	0.191	0.084	**0.075**	**0.060**	0.172	0.079	**0.060**
6	0.037	0.170	0.018	**0.005**	0.074	0.188	0.087	**0.065**	**0.052**	0.171	0.082	0.065
7	0.036	0.161	0.018	**0.004**	0.077	0.182	0.090	**0.065**	**0.049**	0.162	0.086	0.069
8	0.035	0.158	0.020	**0.004**	0.081	0.186	0.092	**0.063**	**0.047**	0.166	0.090	0.074
9	0.032	0.161	0.015	**0.003**	0.082	0.176	0.090	**0.067**	**0.049**	0.162	0.092	0.083
10	0.031	0.157	0.018	**0.003**	0.078	0.181	0.094	**0.067**	**0.050**	0.159	0.092	0.079
20	0.030	0.141	0.028	**0.001**	0.085	0.162	0.102	**0.064**	**0.062**	0.145	0.088	0.068
30	0.030	0.131	0.029	**0.001**	0.085	0.155	0.108	**0.070**	**0.058**	0.139	0.093	0.066
40	0.031	0.118	0.031	**0.000**	0.084	0.142	0.105	**0.078**	**0.053**	0.127	0.094	0.069
50	0.031	0.119	0.029	**0.001**	0.083	0.135	0.107	**0.078**	**0.049**	0.126	0.101	0.062
Avg.	0.041	0.157	0.021	**0.002**	0.087	0.179	0.085	**0.063**	0.065	0.167	0.079	**0.059**
Min.	0.030	0.118	0.015	**0.000**	0.074	0.135	0.019	**0.005**	0.047	0.126	0.022	**0.005**

Boldface numbers indicate the minimum average of classification error rates (the highest accuracy) achieved with the corresponding classifier at each size of selected gene sets, reported in the first column.

**Table 3:** **Average classification error rates yielded by Random Forest,** ***k*** **Nearest Neighbors and Support Vector Machine classifiers on ‘GSE24514’ dataset over all the 50 repetitions of 10‐fold cross validation**

	RF				***k***NN				SVM			
N. genes	Wil‐RS	mRMR	MP	POS	Wil‐RS	mRMR	MP	POS	Wil‐RS	mRMR	MP	POS
1	0.163	0.352	0.182	**0.090**	0.125	0.304	0.147	**0.096**	0.116	0.274	0.141	**0.085**
2	0.108	0.267	0.143	**0.082**	0.086	0.249	0.117	**0.074**	0.085	0.250	0.108	**0.080**
3	0.098	0.219	0.116	**0.068**	0.077	0.223	0.093	**0.068**	0.075	0.215	0.087	**0.067**
4	0.079	0.186	0.121	**0.067**	0.078	0.186	0.082	**0.065**	0.068	0.185	0.077	**0.063**
5	0.074	0.166	0.103	**0.059**	0.072	0.166	0.070	**0.063**	**0.062**	0.166	0.071	**0.062**
6	0.067	0.147	0.090	**0.058**	0.066	0.155	0.068	**0.059**	**0.060**	0.149	0.064	**0.060**
7	0.065	0.137	0.074	**0.058**	**0.059**	0.142	0.064	0.060	**0.059**	0.135	0.061	0.061
8	0.064	0.128	0.068	**0.052**	**0.057**	0.133	0.060	0.058	0.056	0.126	0.057	**0.054**
9	0.063	0.115	0.075	**0.055**	**0.052**	0.127	0.061	0.057	0.053	0.113	0.052	**0.050**
10	0.063	0.104	0.066	**0.051**	**0.048**	0.116	0.058	0.058	0.050	0.105	**0.047**	0.048
20	0.058	0.076	0.047	**0.037**	**0.032**	0.088	0.048	0.050	0.044	0.078	0.041	**0.039**
30	0.057	0.067	0.039	**0.034**	**0.035**	0.071	0.041	0.043	0.042	0.070	0.038	**0.034**
40	0.057	0.073	0.040	**0.034**	**0.037**	0.063	**0.037**	0.042	0.041	0.069	**0.037**	**0.037**
50	0.055	0.063	0.038	**0.032**	**0.036**	0.041	**0.036**	0.039	0.041	0.059	0.038	**0.036**
Avg.	0.077	0.150	0.086	**0.055**	0.061	0.147	0.070	**0.059**	0.061	0.142	0.066	**0.055**
Min.	0.055	0.063	0.038	**0.032**	**0.032**	0.041	0.036	0.039	0.041	0.059	0.037	**0.034**

Boldface numbers indicate the minimum average of classification error rates (the highest accuracy) achieved with the corresponding classifier at each size of selected gene sets, reported in the first column.

The proportional overlapping scores (POS) approach yields a good performance with different classifiers on all datasets. For the Random Forest classifier, in particular on Leukaemia, Breast, GSE24514 and GSE4045 datasets, the classification average error rates on the test sets are less than all other feature selection techniques at all selected genes set sizes. On the Srbct, All and Lung datasets, the POS method provides lower error rates than all other methods on most set sizes. While, on the Prostate dataset, POS shows a comparable performance with the best technique (MP). On the Carcinoma dataset, Wil‐RS technique has outperformed all methods for feature set sizes which are more than 20 genes, whereas for smaller sets, the MP method was the best. More details of the RF classifier’s results can be found in the Additional file 1.

For the *k*NN classifier, POS provides a good classification performance. Its classification average error rates are less than all other compared methods on Leukaemia and Breast datasets for most selected set sizes, see Table 2 and Figure 4. A similar case has been observed in the Lung dataset, see Additional file 2: Table S3. On the GSE24514 dataset, Wil‐RS technique has outperformed all methods for set sizes that are more than eight, whereas for smaller sets, the POS was the best. While, on Srbct and GSE4045 datasets, POS shows a comparable and a worse performance respectively compared with the best techniques, MP and Wil‐RS respectively. More details of the *k*NN classifier’s results can be found in the Additional file 2.

**Figure 5:**
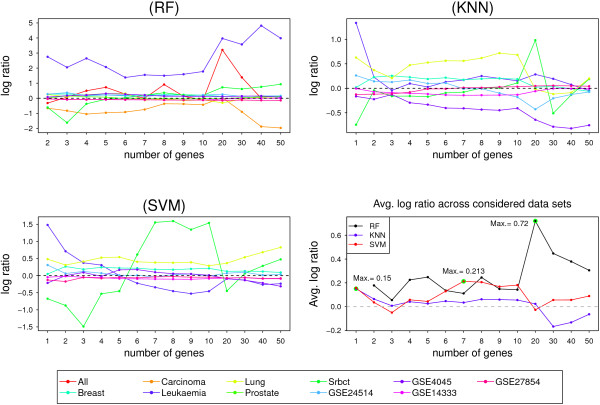
**Log ratio between the error rates of the best compared method and the POS.** Log ratios measure the improvement/deterioration achieved by the proposed method over the best compared method for three different classifiers; RF, *k*NN and SVM. The last panel shows the averages of log ratios across all datasets for each classifier.

For the SVM classifier, POS provides a good classification performance on all used datasets. In particular on Breast and Lung datasets, the classification average error rates on the test sets are less than all other feature selection techniques at all selected genes set sizes, see Figure 4 in the manuscript and Additional file 3: Table S3. The performance of POS outperformed all other compared methods on the GSE24514 and Srbct datasets for almost all feature set sizes, see Table 3 and Figure 4. On Leukaemia and GSE4045 datasets, POS is outperformed by other methods for set sizes more than five and 20 respectively. More details of the SVM classifier’s results can be found in the Additional file 3.The improvement/deterioration in the classification accuracy is analyzed in order to investigate the quality performance of our proposal against the other techniques when the size of the selected gene set varies. The log ratio between the misclassification error rates of the candidate set selected by the best method of the compared techniques and the POS method is separately computed for each classifier on different set sizes up to 50 genes. At each set size, the best method of the compared techniques is identified and the log ratio between its error rate and corresponding error rate of the POS method is reported. Figure 5 shows the results with each classifier. Positive values indicate improvements of a classification performance achieved by the POS method over the second best technique. The panel on right bottom of Figure 5 shows the averages of log ratios across all considered datasets for each classifier.

The POS approach provides improvements over the best method of the compared techniques for most datasets with all classifiers, see panels of RF, *k*NN and SVM in Figure 5. On average across all datasets, POS achieves an improvement over the best compared techniques at all set sizes for RF classifier by between 0.055 and 0.720, measured by the log ratio of the error rates. The highest improvement in RF classification performance measured by log ratio, 0.720, is obtained at gene sets of size 20. For smaller sizes, the performance ratio decreases, but the POS approach still provides the best accuracy, see Figure 5. For *k*NN and SVM classifiers, the averages of improvements across Leukaemia, Breast, Srbct, Lung, GSE24514, GSE4045, GSE14333 and GSE27854 have been depicted at different set sizes up to 50 genes. The proposed approach achieves improvements for *k*NN classifier at set sizes not more than 20 features. The highest improvement measured by log ratio, 0.150, is obtained at the selected sets composed of a single gene. For SVM classifier, improvements over the best method of the compared techniques are achieved by the POS method at most set sizes. The highest improvement measured by the log ratio of the error rates, 0.213, is observed at gene sets of size seven, see the right bottom panel of Figure 5.

The best performing technique among the compared methods is not always the same for neither all selected gene set sizes, all datasets nor all classifiers. Hence, the POS algorithm could keep its better performance for large as well as small sets of selected genes with Random Forest and Support Vector Machine classifiers on individual datasets. While it could keep its best performance with *k* Nearest Neighbor classifier for only feature sets with small sizes (specifically, not more than 20). Consequently, the POS feature selection approach is more able to adapt to different pattern of data and to different classifiers than the other techniques, whose performance is more affected by varying the data characteristics and the used classifier.

A method which is more able to minimize the dependency within its selected candidates can reach a particular level of accuracy using a smaller set of genes. To highlight the entire performances of the compared methods against our proposed approach, we also performed a comparison between the minimum error rates achieved by each method. Each method obtains its particular minimum at different size of selected gene set. Tables 4, 5 and 6 summarizes these results for RF, *k*NN and SVM classifiers respectively. Each row shows the minimum error rate (along‐with its corresponding size, shown in brackets) obtained by all methods for a specific dataset, reported in the first column. Since the inherent principal of the ISIS method may result in selecting sets with different sizes for each fold of the cross validation, the estimated error rate has been reported along‐with the average size of the selected feature sets, shown in brackets. In addition, the error rates of the corresponding classifier with the full set of features, without feature selection, are reported in the last column of Tables 4, 5 and 6. A similar comparison scheme is performed in [39].

**Table 4:** **The minimum error rates yielded by Random Forest classifier with feature selection methods along‐with the classification error without selection**

Dataset	ISIS	Wil‐RS	mRMR	MP	POS	Full set
Leukaemia	0.003 (1)	0.030 (20)	0.118 (40)	0.015 (9)	**0.0002** (40)	0.049
Breast	0.407 (4)	0.371 (50)	0.407 (48)	0.354 (48)	**0.308** (45)	0.369
Srbct	0.092 (2.63)	0.069 (24)	0.074 (46)	0.009 (32)	**0.003** (48)	0.0008
Prostate	0.097 (4.18)	0.200 (50)	0.140 (50)	0.069 (50)	**0.062** (50)	0.088
All	0.0004 (1.018)	0.143 (40)	0.011 (50)	**0** (40)	**0** (20)	0
Lung	0.022 (3.26)	0.040 (30)	0.016 (48)	0.008 (46)	**0.007** (48)	0.003
Carcinoma	0.171 (1.29)	**0.003** (41)	0.017 (44)	0.019 (5)	0.026 (20)	0.027
GSE24514	0.107 (1.96)	0.054 (47)	0.063 (50)	0.036 (48)	**0.032** (24)	0.041
GSE4045	0.27 (1.47)	0.134 (24)	0.187 (37)	0.137 (21)	**0.114** (27)	0.205
GSE14333	0.423 (9)	**0.421** (10)	‐	0.438 (31)	0.437 (34)	0.414
GSE27854	0.448 (5)	**0.401** (15)	‐	0.444 (49)	0.451 (6)	0.488

The numbers in brackets represent the size, average size for ISIS method, of the gene set that corresponding to the minimum error rate. Boldface numbers indicate the lowest error rate (the highest accuracy) among the compared methods for the corresponding datasets.

**Table 5:** **The minimum error rates yielded by** ***k*** **Nearest Neighbor classifier with feature selection methods along‐with the classification error without selection**

Dataset	ISIS	Wil‐RS	mRMR	MP	POS	Full set
Leukaemia	0.064 (1)	0.074 (6)	0.135 (50)	0.019 (1)	**0.005** (1)	0.109
Breast	0.385 (4)	0.405 (11)	0.404 (50)	0.346 (19)	**0.332** (11)	0.405
Srbct	0.105 (2.63)	0.157 (3)	0.098 (48)	**0.005** (26)	**0.005** (22)	0.034
Lung	0.030 (3.26)	0.203 (12)	0.027 (49)	0.017 (17)	**0.011** (12)	0.0005
GSE24514	0.074 (1.96)	**0.032** (20)	0.041 (50)	0.036 (50)	0.039 (50)	0.041
GSE4045	0.239 (1.47)	**0.066** (43)	0.207 (38)	0.137 (50)	0.142 (3)	0.103
GSE14333	0.425 (9)	**0.420** (8)	‐	0.455 (23)	0.450 (34)	0.438
GSE27854	0.432 (5)	**0.420** (3)	‐	0.454 (13)	**0.420** (6)	0.464

The numbers in brackets represent the size, average size for ISIS method, of the gene set that corresponding to the minimum error rate. Boldface numbers indicate the lowest error rate (the highest accuracy) among the compared methods for the corresponding datasets.

**Table 6:** **The minimum error rates yielded by Support Vector Machine classifier with feature selection methods along‐with the classification error without selection**

Dataset	ISIS	Wil‐RS	mRMR	MP	POS	Full set
Leukaemia	0.018 (1)	0.047 (8)	0.126 (50)	0.022 (1)	**0.005** (1)	0.131
Breast	0.409 (4)	0.401 (39)	0.407 (50)	0.359 (21)	**0.313** (22)	0.438
Srbct	0.106 (2.63)	0.131 (50)	0.124 (49)	0.010 (21)	**0.003** (8)	0.079
Lung	0.013 (3.26)	0.066 (50)	0.026 (50)	0.021 (19)	**0.010** (47)	0.024
GSE24514	0.090 (1.96)	0.041 (40)	0.059 (50)	0.037 (40)	**0.034** (30)	0.070
GSE4045	0.236 (1.47)	0.134 (24)	0.187 (37)	**0.095** (47)	0.114 (29)	0.214
GSE14333	0.416 (9)	0.427 (9)	‐	**0.412** (1)	0.431 (1)	0.407
GSE27854	0.434 (5)	**0.431** (25)	‐	0.465 (13)	0.456 (8)	0.50

The numbers in brackets represent the size, average size for ISIS method, of the gene set that corresponding to the minimum error rate. Boldface numbers indicate the lowest error rate (the highest accuracy) among the compared methods for the corresponding datasets.

An effective feature selection technique is expected to produce stable outcomes across several sub‐samples of the considered dataset. This property is particularly desirable for biomarker selections within a diagnostic setting. A stable feature selection method should yield a set of biological informative markers that are selected quite often, and randomly chosen features that are selected rarely or never.

**Figure 6:**
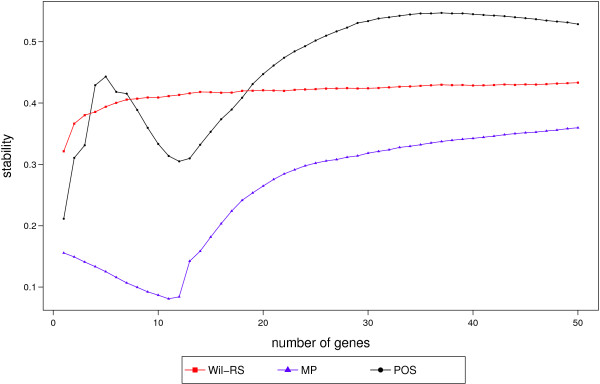
**Stability scores for ‘GSE27854’ dataset.** Stability scores at different sizes of features sets that selected by Wil‐RS, MP and POS methods on ‘GSE27854’ dataset.

**Figure 7:**
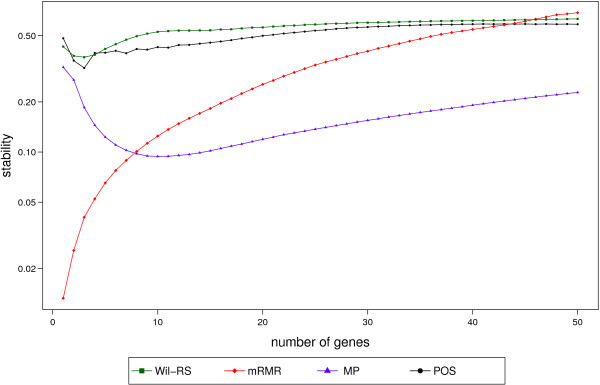
**Stability scores for ‘GSE24514’ dataset.** Stability scores at different sizes of features sets that selected by Wil‐RS, mRMR, MP and POS methods on ‘GSE24514’ dataset.

The stability index proposed by Lausser et al. [40] is used to measure the stability of the compared method at different set sizes of features. Values of this stability score range from 1/*λ*, where *λ* is the total number of used sub‐samples (in our context, *λ*=500), for the worst unstable selections to 1 for the full stable selection. Table 7 and Figures 6 and 7 show the stability scores of different feature selection methods for the ‘Srbct’, ‘GSE27854’ and ‘GSE24514’ datasets respectively. Figure 6 shows that our proposed approach provides more stable feature selections than Wil‐RS and MP methods at most set sizes selected from ‘GSE27854’ dataset. For GSE24514 dataset, Figure 7 depicts the stability scores of compared feature selection techniques at different set sizes. Unlike the mRMR and the MP approaches, both the Wil‐RS and the POS methods keep their stability degree for different sizes of feature sets. The POS method provides a stability degree close to the well established Wil‐RS method. For the ‘Srbct’ data, the best stability scores among the compared methods are yielded by POS at most set sizes, see Table 7.

**Table 7:** **Stability scores of the feature selection techniques over 50 repetitions of 10‐fold cross validation for ‘Srbct’ dataset**

N. selected genes	Wil‐RS	mRMR	MP	POS
5	0.789	0.097	0.815	0.760
10	0.804	0.198	0.788	0.844
15	0.804	0.302	0.853	0.911
20	0.857	0.405	0.898	0.908
25	0.883	0.506	0.871	0.872
30	0.896	0.579	0.871	0.870
35	0.868	0.640	0.852	0.859
40	0.858	0.705	0.833	0.847
45	0.862	0.754	0.812	0.835
50	0.873	0.803	0.800	0.820

**Figure 8:**
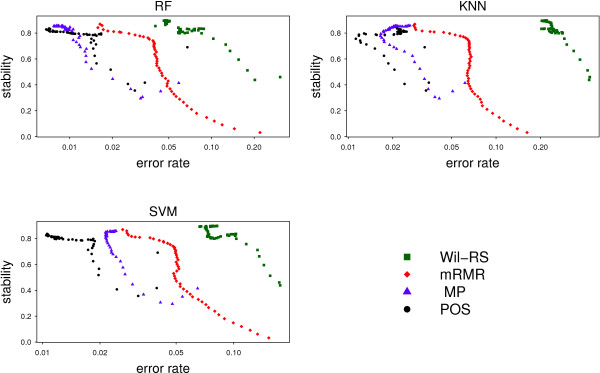
**Stability‐accuracy plot for ‘Lung’ dataset.** The stability of the feature selection methods against the corresponding estimated error rates on ‘Lung’ dataset. The error rates have been measured by 50 repetations of 10‐fold cross validation for three different classifiers: Random Forest (RF); *k* Nearest Neighbor (*k*NN); Support Vector Machine (SVM).

**Figure 9:**
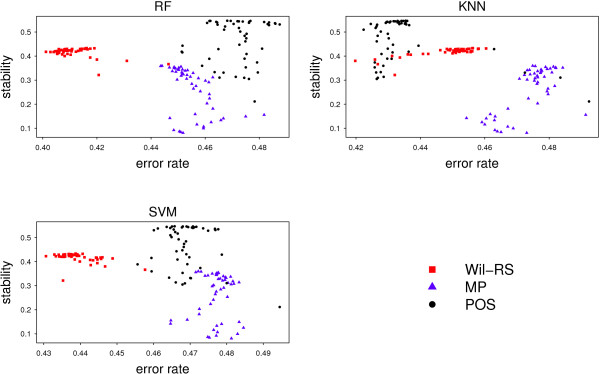
**Stability‐accuracy plot for ‘GSE27854’ dataset.** The stability of the feature selection methods against the corresponding estimated error rates on ‘GSE27854’ dataset. The error rates have been measured by 50 repetations of 10‐fold cros validation for three different classifiers: Random Forest (RF); *k* Nearest Neighbor (*k*NN); Support Vector Machine (SVM).

A stable selection does not guarantee the relevancy of the selected features to the considered response of the target class labels. The prediction accuracy yielded by a classifier based on the selected features should also be highlighted. The relation between the accuracy and stability has been outlined by Figures 8 and 9 for the ‘Lung’ and ‘GSE27854’ respectively. The stability scores were combined with corresponding error rates yielded by three different classifiers: RF; *k*NN; SVM. Different dots for the same feature selection method correspond to different set sizes of features. Since stability degree increases from the bottom to the top on the vertical axis and the classification error increases to the right on the horizontal axis, the best method is the one whose dots are depicted in the upper‐left corner of the plot. For all classifiers, our proposed method achieve a good trade‐off between accuracy and stability for ‘Lung’ data, see Figure 8. For ‘GSE27854’ data with the *k*NN classifier, POS provides a better trade‐off between accuracy and stability than other compared methods. Whereas with the RF and SVM classifiers, POS is outperformed by Wil‐RS.

Genomic experiments are representative examples for high‐dimensional datasets. However, our proposal of feature selection can be also used on other high‐dimensional data, e.g. [41] and [42].

All procedures described in this manuscript have been programmed into an R package named ‘propOverlap’. It would be available for download from the Comprehensive R Archive Network (CRAN) repository (http://cran.us.r-project.org/) as soon as possible.

## Conclusion

The idea of selecting genes based on analysing the overlap of their expressions across two phenotypes, taking into account the proportions of overlapping samples, is considered in this article. To this end, we defined core gene expressions and robustly constructed gene masks that allow us to report a gene’s predictive power avoiding the effects of outliers. In addition, a novel score, named as the Proportional Overlapping Score (*POS*), is proposed by which a gene’s overlapping degree is estimated. We then utilized the constructed gene masks along‐with the gene scores to assign the minimum subset of genes that provide the maximum number of correctly classified samples in a training set. This minimum subset of genes is then combined with the top ranked genes according to the *POS* to produce a final gene selection.

Our new procedure is applied on eleven publicly available gene expression datasets with different characteristics. Feature sets of different sizes, up to 50 genes, are selected using widely used gene selection methods: Wilcoxon Rank Sum (Wil‐RS); Minimum redundancy maximum relevance (mRMR); MaskedPainter (MP); Iteratively sure independence screening (ISIS) along‐with our proposal, POS. Then, the prediction models of three different classifiers: Random Forest; *k* Nearest Neighbor; Support Vector Machine are constructed with the selected features. The estimated classification error rates obtained by the considered classifiers are used for evaluating the performance of POS.

For the Random Forest classifier, POS performed better than the compared feature selection methods on ‘Leukaemia’, ‘Breast’, ‘GSE24514’ and ‘GSE4045’ datasets at all gene set sizes that have been investigated. POS also outperformed all other methods on ‘Lung’, ‘All’ and ‘Srbct’ datasets at: small (i.e., less than 7); moderate and large (i.e., >2); large (i.e., >5) sets of genes respectively. On average, our proposal improves the compared techniques by between 5% and 51% of the misclassification error rates achieved by their candidates.

For the *k* Nearest Neighbor classifier, POS outperformed all other methods on ‘Leukaemia’, ‘Breast’, ‘Lung’ and ‘GSE27854’. While it shows a comparable performance to the MaskedPainter method on the ‘Srbct’. On average across all considered datasets, POS approach improves the best performance of the compared methods by up to 20% of the misclassification error rates achieved using their selections at small set sizes less than 20 features.

For the Support Vector Machine classifier, POS outperformed all other methods on ‘Leukaemia’, ‘Breast’, ‘Srbct’, ‘Lung’ and ‘GSE24514’. While the MaskedPainter provides the minimum error rates on ‘GSE4045’ and ‘GSE14333’. Whereas on ‘GSE27854’ data, the Wilcoxon Rank Sum is the best. On average across all considered datasets, POS approach improves the best performance of the compared methods by up to 26% of the misclassification error rates achieved using their selections at different set sizes.

The stability of the selections yielded by the compared feature selection methods using the cross validation technique has been highlighted. Stability scores computed at different set sizes of the selected features show that the proposed method has a stable performance for different sizes of selected features. The analysed relationship between classification accuracies yielded by three different classifiers and stability confirms that the POS method can provide a good trade‐off between stability and classification accuracy.

The intuition for the better performance of our new method might be that when incorporating together genes with less overlapping degrees across different phenotypes, estimated by taking into account a useful element of overlapping analysis, i.e. the proportions of overlapped samples, with those genes which could capture the distinct underlying structure of samples by means of gene masks, then a classifier could be more able to gain more information from the learning process than that of those could be gained by other selected same sized sets of genes.

In the future, one can investigate the possibility of extending POS method to handle multi‐class situations. Constructing a framework for POS in which mutual information between genes are considered in the final gene set might be another useful direction. Such a framework could be effective in selecting the discriminative genes with a low degree of dependency.

## Availability of supporting data

The datasets supporting the results of this article are publicly available. The Lung and Leukaemia datasets can be downloaded from [http://cilab.ujn.edu.cn/datasets.htm]. The Srbct and Prostate datasets are available in [http://www.gems-system.org/]. The Carcinoma dataset can be found in [http://genomics-pubs.princeton.edu/oncology/]. While the Colon, All and Breast datasets are available in the [Bioconductor] repository, [http://www.bioconductor.org/] from the R packages [‘ColonCA’, ‘All’ and ‘cancerdata’ respectively]. Other datasets are available in the [Gene Expression Omnibus (GEO)] repository [http://www.ncbi.nlm.nih.gov/geo/][accession id’s: GSE24514; GSE4045; GSE14333; GSE27854].

## Electronic supplementary material

Additional file 1: **Classification error rates obtained by Random Forest Classifier.** Average classification error rates yielded by the Random Forest classifier using Wilcoxon rank sum (Wil‐RS), Minimum redundancy maximum relevance (mRMR), MaskedPainter (MP) and proportional overlapping scores (POS) feature selection techniques on ‘Breast’, ‘Srbct’, ‘Prostate’, ‘All’, ‘Lung’, ‘Carcinoma’, ‘GSE4045’, ‘GSE14333’ and ‘GSE27854’ datasets over 50 repetitions of 10‐fold cross validation are presented in nine tables, a table for each dataset. Each row provides the average classification error rate at a specific number of selected genes (reported in the first column). (PDF 16 KB)

Additional file 2: **Classification error rates obtained by** ***k*** **Nearest Neighbor Classifier.** Average classification error rates yielded by the *k* Nearest Neighbor classifier using Wilcoxon rank sum (Wil‐RS), Minimum redundancy maximum relevance (mRMR), MaskedPainter (MP) and proportional overlapping scores (POS) feature selection techniques on ‘Breast’, ‘Srbct’, ‘Lung’, ‘GSE4045’, ‘GSE14333’ and ‘GSE27854’ datasets over 50 repetitions of 10‐fold cross validation are presented in six tables, a table for each dataset. Each row provides the average classification error rate at a specific number of selected genes (reported in the first column). (PDF 14 KB)

Additional file 3: **Classification error rates obtained by Support Vector Machine Classifier.** Average classification error rates yielded by the Support Vector Machine classifier using Wilcoxon rank sum (Wil‐RS), Minimum redundancy maximum relevance (mRMR), MaskedPainter (MP) and proportional overlapping scores (POS) feature selection techniques on ‘Breast’, ‘Srbct’, ‘Lung’, ‘GSE4045’, ‘GSE14333’ and ‘GSE27854’ datasets over 50 repetitions of 10‐fold cross validation are presented in six tables, a table for each dataset. Each row provides the average classification error rate at a specific number of selected genes (reported in the first column). (PDF 15 KB)
