# Supplementary material for: A feature selection method for classification within functional genomics experiments based on the proportional overlapping score
Source: BMC Bioinformatics. 2014 Aug 11;15(1):274. doi: 10.1186/1471-2105-15-274 (PMC4141116; doi:10.1186/1471-2105-15-274)
Supplement: Supplementary file 1 — Additional file 1: Classification error rates obtained by Random Forest Classifier. Average classification error rates yielded by the Random Forest classifier using Wilcoxon rank sum (Wil‐RS), Minimum redundancy maximum relevance (mRMR), MaskedPainter (MP) and proportional overlapping scores (POS) feature selection techniques on ‘Breast’, ‘Srbct’, ‘Prostate’, ‘All’, ‘Lung’, ‘Carcinoma’, ‘GSE4045’, ‘GSE14333’ and ‘GSE27854’ datasets over 50 repetitions of 10‐fold cross validation are presented in nine tables, a table for each dataset. Each row provides the average classification error rate at a specific number of selected genes (reported in the first column). (PDF 16 KB) [file 12859_2014_6543_MOESM1_ESM.pdf]

Additional file 1: RF error rates for different feature selection methods

Additional file 1: Table S1: 'Breast' dataset

N.genes	Wil-RS	mRMR	MP	POS
2	0.437	0.483	0.499	0.333
3	0.439	0.475	0.474	0.330
4	0.433	0.471	0.463	0.349
5	0.428	0.474	0.453	0.342
6	0.422	0.462	0.443	0.350
7	0.421	0.458	0.436	0.340
8	0.426	0.458	0.433	0.336
9	0.418	0.459	0.432	0.331
10	0.417	0.459	0.425	0.338
20	0.396	0.448	0.374	0.331
30	0.375	0.442	0.367	0.318
40	0.384	0.425	0.365	0.317
50	0.371	0.407	0.357	0.310

Additional file 1: Table S2: 'Srbct' dataset

N.genes	Wil-RS	mRMR	MP	POS
2	0.083	0.414	0.046	0.085
3	0.095	0.369	0.013	0.064
4	0.103	0.335	0.029	0.041
5	0.081	0.315	0.026	0.028
6	0.079	0.281	0.026	0.025
7	0.088	0.272	0.028	0.022
8	0.091	0.246	0.026	0.018
9	0.092	0.229	0.027	0.022
10	0.097	0.222	0.026	0.022
20	0.085	0.153	0.012	0.006
30	0.077	0.097	0.011	0.006
40	0.081	0.095	0.012	0.006
50	0.090	0.077	0.014	0.006

Additional file 1: Table S3: 'Prostate' dataset

N.genes	Wil-RS	mRMR	MP	POS
2	0.435	0.415	0.117	0.117
3	0.481	0.390	0.098	0.083
4	0.470	0.366	0.097	0.088
5	0.452	0.345	0.092	0.086
6	0.433	0.324	0.089	0.091
7	0.432	0.313	0.087	0.088
8	0.433	0.294	0.090	0.092
9	0.445	0.274	0.088	0.092
10	0.440	0.265	0.085	0.088
20	0.218	0.192	0.070	0.071
30	0.215	0.157	0.071	0.068
40	0.216	0.158	0.071	0.065
50	0.200	0.140	0.069	0.062

Additional file 1: Table S4: 'All' dataset

N.genes	Wil-RS	mRMR	MP	POS
2	0.314	0.315	0.020	0.027
3	0.295	0.287	0.012	0.011
4	0.284	0.284	0.016	0.010
5	0.269	0.269	0.010	0.005
6	0.262	0.261	0.007	0.005
7	0.262	0.250	0.006	0.006
8	0.260	0.239	0.005	0.002
9	0.272	0.236	0.006	0.005
10	0.274	0.229	0.005	0.005
20	0.246	0.114	0.002	0.000
30	0.225	0.036	0.001	0.000
40	0.143	0.024	0.000	0.000
50	0.152	0.011	0.000	0.000

Additional file 1: Table S5: 'Lung' dataset

N.genes	Wil-RS	mRMR	MP	POS
2	0.202	0.144	0.043	0.034
3	0.179	0.120	0.032	0.029
4	0.155	0.106	0.033	0.026
5	0.135	0.092	0.026	0.019
6	0.126	0.079	0.021	0.015
7	0.103	0.071	0.014	0.016
8	0.085	0.067	0.013	0.013
9	0.081	0.062	0.013	0.015
10	0.081	0.059	0.013	0.014
20	0.068	0.044	0.010	0.013
30	0.040	0.041	0.009	0.009
40	0.047	0.031	0.008	0.008
50	0.048	0.016	0.008	0.007

Additional file 1: Table S6: 'Carcinoma' dataset

N.genes	Wil-RS	mRMR	MP	POS
2	0.472	0.347	0.038	0.073
3	0.508	0.280	0.025	0.056
4	0.290	0.243	0.022	0.063
5	0.270	0.221	0.019	0.049
6	0.120	0.199	0.022	0.056
7	0.114	0.182	0.023	0.049
8	0.097	0.157	0.025	0.036
9	0.095	0.140	0.026	0.038
10	0.081	0.131	0.023	0.035
20	0.028	0.065	0.027	0.026
30	0.011	0.039	0.025	0.027
40	0.004	0.023	0.026	0.027
50	0.004	0.021	0.027	0.029

Additional file 1: Table S7: 'GSE4045' dataset

N.genes	Wil-RS	mRMR	MP	POS
2	0.201	0.266	0.208	0.186
3	0.195	0.245	0.180	0.152
4	0.193	0.236	0.194	0.156
5	0.178	0.223	0.172	0.126
6	0.182	0.228	0.169	0.129
7	0.177	0.218	0.160	0.130
8	0.176	0.213	0.155	0.136
9	0.172	0.217	0.151	0.132
10	0.172	0.211	0.147	0.132
20	0.143	0.193	0.141	0.125
30	0.140	0.197	0.149	0.122
40	0.141	0.192	0.152	0.121
50	0.143	0.192	0.154	0.134

Additional file 1: Table S8: 'GSE14333' dataset

N.genes	Wil-RS	MP	POS
2	0.454	0.491	0.474
3	0.440	0.485	0.481
4	0.439	0.490	0.476
5	0.435	0.483	0.478
6	0.428	0.475	0.474
7	0.426	0.472	0.474
8	0.424	0.472	0.475
9	0.423	0.471	0.475
10	0.421	0.471	0.474
20	0.438	0.460	0.461
30	0.449	0.438	0.445
40	0.453	0.445	0.441
50	0.452	0.442	0.445

Additional file 1: Table S9: ‘GSE27854’ dataset

N.genes	Wil-RS	MP	POS
2	0.446	0.475	0.465
3	0.431	0.467	0.467
4	0.420	0.466	0.465
5	0.418	0.462	0.452
6	0.408	0.457	0.451
7	0.410	0.459	0.452
8	0.407	0.459	0.462
9	0.405	0.448	0.459
10	0.409	0.450	0.463
20	0.405	0.456	0.473
30	0.414	0.451	0.471
40	0.408	0.450	0.472
50	0.416	0.444	0.484
