# Supplementary material for: A feature selection method for classification within functional genomics experiments based on the proportional overlapping score
Source: BMC Bioinformatics. 2014 Aug 11;15(1):274. doi: 10.1186/1471-2105-15-274 (PMC4141116; doi:10.1186/1471-2105-15-274)
Supplement: Supplementary file 2 — Additional file 2: Classification error rates obtained by k Nearest Neighbor Classifier. Average classification error rates yielded by the k Nearest Neighbor classifier using Wilcoxon rank sum (Wil‐RS), Minimum redundancy maximum relevance (mRMR), MaskedPainter (MP) and proportional overlapping scores (POS) feature selection techniques on ‘Breast’, ‘Srbct’, ‘Lung’, ‘GSE4045’, ‘GSE14333’ and ‘GSE27854’ datasets over 50 repetitions of 10‐fold cross validation are presented in six tables, a table for each dataset. Each row provides the average classification error rate at a specific number of selected genes (reported in the first column). (PDF 14 KB) [file 12859_2014_6543_MOESM2_ESM.pdf]

Additional file 2: kNN error rates for different feature selection methods

Additional file 2: Table S1: 'Breast' dataset

N.genes	Wil-RS	mRMR	MP	POS
1	0.425	0.486	0.510	0.417
2	0.463	0.474	0.480	0.368
3	0.471	0.465	0.468	0.360
4	0.457	0.452	0.447	0.356
5	0.467	0.456	0.434	0.360
6	0.470	0.453	0.432	0.348
7	0.470	0.450	0.418	0.351
8	0.454	0.449	0.415	0.342
9	0.451	0.441	0.417	0.340
10	0.441	0.439	0.406	0.337
20	0.430	0.423	0.348	0.340
30	0.438	0.431	0.354	0.341
40	0.417	0.413	0.359	0.362
50	0.438	0.404	0.354	0.377

Additional file 2: Table S2: 'Srbct' dataset

N.genes	Wil-RS	mRMR	MP	POS
1	0.422	0.452	0.064	0.135
2	0.180	0.435	0.094	0.098
3	0.157	0.398	0.054	0.063
4	0.185	0.376	0.043	0.050
5	0.189	0.361	0.043	0.051
6	0.252	0.345	0.042	0.046
7	0.299	0.332	0.040	0.044
8	0.364	0.321	0.041	0.040
9	0.394	0.308	0.041	0.040
10	0.424	0.308	0.041	0.037
20	0.367	0.265	0.018	0.007
30	0.383	0.246	0.005	0.008
40	0.477	0.192	0.008	0.009
50	0.460	0.099	0.011	0.009

Additional file 2: Table S3: 'Lung' dataset

N.genes	Wil-RS	mRMR	MP	POS
1	0.430	0.162	0.062	0.033
2	0.426	0.139	0.051	0.035
3	0.416	0.125	0.041	0.034
4	0.370	0.112	0.039	0.024
5	0.335	0.105	0.035	0.021
6	0.301	0.095	0.033	0.019
7	0.290	0.087	0.031	0.017
8	0.281	0.081	0.028	0.015
9	0.257	0.080	0.027	0.013
10	0.234	0.078	0.024	0.012
20	0.239	0.064	0.017	0.018
30	0.239	0.065	0.020	0.022
40	0.217	0.057	0.023	0.025
50	0.213	0.028	0.026	0.021

Additional file 2: Table S4: 'GSE4045' dataset

N.genes	Wil-RS	mRMR	MP	POS
1	0.180	0.235	0.227	0.213
2	0.132	0.228	0.172	0.165
3	0.125	0.231	0.153	0.142
4	0.124	0.227	0.144	0.166
5	0.115	0.225	0.148	0.160
6	0.115	0.231	0.153	0.172
7	0.114	0.229	0.147	0.171
8	0.111	0.231	0.149	0.171
9	0.106	0.229	0.148	0.166
10	0.110	0.221	0.150	0.165
20	0.083	0.211	0.147	0.157
30	0.070	0.221	0.147	0.154
40	0.070	0.209	0.144	0.158
50	0.078	0.214	0.137	0.166

Additional file 2: Table S5: 'GSE14333' dataset

N.genes	Wil-RS	MP	POS
1	0.469	0.472	0.472
2	0.443	0.480	0.476
3	0.434	0.488	0.474
4	0.431	0.488	0.478
5	0.429	0.489	0.482
6	0.422	0.487	0.483
7	0.419	0.483	0.483
8	0.419	0.480	0.482
9	0.420	0.475	0.482
10	0.423	0.476	0.483
20	0.443	0.459	0.470
30	0.454	0.468	0.456
40	0.453	0.482	0.456
50	0.453	0.497	0.456

Additional file 2: Table S6: 'GSE27854' dataset

N.genes	Wil-RS	MP	POS
1	0.432	0.491	0.492
2	0.427	0.463	0.483
3	0.420	0.460	0.472
4	0.426	0.463	0.463
5	0.434	0.459	0.439
6	0.431	0.460	0.436
7	0.437	0.467	0.431
8	0.436	0.469	0.431
9	0.441	0.463	0.428
10	0.442	0.464	0.428
20	0.453	0.480	0.431
30	0.449	0.476	0.425
40	0.454	0.474	0.432
50	0.456	0.475	0.437
