# Supplementary material for: A feature selection method for classification within functional genomics experiments based on the proportional overlapping score
Source: BMC Bioinformatics. 2014 Aug 11;15(1):274. doi: 10.1186/1471-2105-15-274 (PMC4141116; doi:10.1186/1471-2105-15-274)
Supplement: Supplementary file 3 — Additional file 3: Classification error rates obtained by Support Vector Machine Classifier. Average classification error rates yielded by the Support Vector Machine classifier using Wilcoxon rank sum (Wil‐RS), Minimum redundancy maximum relevance (mRMR), MaskedPainter (MP) and proportional overlapping scores (POS) feature selection techniques on ‘Breast’, ‘Srbct’, ‘Lung’, ‘GSE4045’, ‘GSE14333’ and ‘GSE27854’ datasets over 50 repetitions of 10‐fold cross validation are presented in six tables, a table for each dataset. Each row provides the average classification error rate at a specific number of selected genes (reported in the first column). (PDF 15 KB) [file 12859_2014_6543_MOESM3_ESM.pdf]

Additional file 3: SVM error rates for different feature selection methods

Additional file 3: Table S1: 'Breast' dataset

N.genes	Wil-RS	mRMR	MP	POS
1	0.417	0.481	0.488	0.398
2	0.422	0.470	0.458	0.324
3	0.432	0.466	0.445	0.359
4	0.485	0.454	0.432	0.338
5	0.489	0.461	0.424	0.339
6	0.497	0.457	0.415	0.334
7	0.496	0.456	0.410	0.343
8	0.486	0.447	0.407	0.342
9	0.480	0.446	0.404	0.332
10	0.468	0.444	0.405	0.328
20	0.426	0.443	0.363	0.321
30	0.401	0.440	0.366	0.321
40	0.413	0.426	0.365	0.324
50	0.411	0.407	0.361	0.329

Additional file 3: Table S2: 'Srbct' dataset

N.genes	Wil-RS	mRMR	MP	POS
1	0.421	0.454	0.068	0.134
2	0.176	0.419	0.041	0.099
3	0.174	0.397	0.014	0.060
4	0.152	0.361	0.017	0.029
5	0.143	0.343	0.012	0.018
6	0.149	0.311	0.011	0.006
7	0.150	0.303	0.015	0.003
8	0.165	0.286	0.015	0.003
9	0.184	0.268	0.016	0.004
10	0.180	0.266	0.017	0.004
20	0.177	0.213	0.011	0.018
30	0.152	0.161	0.023	0.022
40	0.162	0.147	0.025	0.019
50	0.131	0.126	0.029	0.018

Additional file 3: Table S3: 'Lung' dataset

N.genes	Wil-RS	mRMR	MP	POS
1	0.173	0.153	0.065	0.040
2	0.175	0.133	0.054	0.040
3	0.155	0.122	0.048	0.032
4	0.144	0.110	0.041	0.025
5	0.136	0.100	0.034	0.020
6	0.133	0.090	0.030	0.020
7	0.116	0.084	0.027	0.018
8	0.103	0.080	0.026	0.018
9	0.102	0.074	0.026	0.017
10	0.103	0.072	0.024	0.018
20	0.081	0.049	0.022	0.015
30	0.073	0.050	0.022	0.013
40	0.080	0.043	0.023	0.012
50	0.066	0.026	0.024	0.011

Additional file 3: Table S4: 'GSE4045' dataset

N.genes	Wil-RS	mRMR	MP	POS
1	0.201	0.330	0.221	0.249
2	0.201	0.266	0.186	0.186
3	0.195	0.245	0.166	0.152
4	0.193	0.236	0.153	0.156
5	0.178	0.223	0.149	0.126
6	0.182	0.228	0.154	0.129
7	0.177	0.218	0.143	0.130
8	0.176	0.213	0.143	0.136
9	0.172	0.217	0.139	0.132
10	0.172	0.211	0.134	0.132
20	0.143	0.193	0.114	0.125
30	0.140	0.197	0.106	0.122
40	0.141	0.192	0.098	0.121
50	0.143	0.192	0.098	0.134

Additional file 3: Table S5: 'GSE14333' dataset

N.genes	Wil-RS	MP	POS
1	0.447	0.412	0.431
2	0.453	0.447	0.455
3	0.440	0.463	0.461
4	0.437	0.465	0.464
5	0.431	0.465	0.471
6	0.429	0.461	0.471
7	0.430	0.454	0.475
8	0.427	0.452	0.474
9	0.427	0.451	0.475
10	0.431	0.454	0.470
20	0.460	0.440	0.472
30	0.468	0.432	0.460
40	0.472	0.423	0.463
50	0.468	0.423	0.462

Additional file 3: Table S6: 'GSE27854' dataset

N.genes	Wil-RS	MP	POS
1	0.435	0.465	0.494
2	0.458	0.483	0.480
3	0.447	0.479	0.470
4	0.443	0.477	0.479
5	0.445	0.485	0.466
6	0.440	0.476	0.470
7	0.443	0.477	0.459
8	0.443	0.483	0.456
9	0.438	0.477	0.459
10	0.445	0.475	0.464
20	0.436	0.477	0.468
30	0.440	0.482	0.466
40	0.434	0.475	0.472
50	0.439	0.472	0.475
